# Clinical Analysis of 42 Cases of Ocular Ischemic Syndrome

**DOI:** 10.1155/2018/2606147

**Published:** 2018-03-11

**Authors:** Jingyi Luo, Zhichao Yan, Yu Jia, Rongjiang Luo

**Affiliations:** ^1^Department of Ophthalmology, The First Affiliated Hospital, Sun Yat-sen University, Guangzhou 510080, China; ^2^State Key Laboratory of Ophthalmology, Zhongshan Ophthalmic Center, Sun Yat-sen University, Guangzhou 510060, China; ^3^Department of Ophthalmology, The Second Affiliated Hospital, Guangzhou Medical University, Guangzhou 510260, China

## Abstract

Ocular ischemic syndrome (OIS) is a severe ocular disease caused by ocular hypoperfusion due to stenosis or occlusion of the common or internal carotid arteries. OIS is easily misdiagnosed or undiagnosed given its asymptomatic onset and complicated ocular manifestations. The present study reviewed 42 patients with OIS, including 30 males (71.43%), 29 older patients (69.05%, >61 yrs), and 35 patients (83.33%) with two or more systemic diseases. Only 6 patients had ocular symptoms as the initial signs upon visiting the Department of Ophthalmology of three hospitals (the First Affiliated Hospital, Sun Yat-sen University; Zhongshan Ophthalmic Center, Sun Yat-sen University; and the Second Affiliated Hospital, Guangzhou Medical University). The ocular symptoms varied from visual deterioration to periorbital pain. Thirty-seven patients (88.10%) complained of constitutional symptoms. Ocular manifestations were diverse and involved both anterior and posterior segments. We reported a case of corneal edema and corneal epithelium erosion in the ipsilateral eye due to internal carotid artery stenosis. As the clinical manifestations of OIS are complex, ophthalmologists must carefully examine patients to avoid a misdiagnosis or a failure to diagnose. The management of OIS requires cooperation with cardiologists and neurologists.

## 1. Introduction

Ocular ischemic syndrome (OIS) comprises a spectrum of ocular characteristics caused by arterial hypoperfusion of the eye. The condition manifests visual deterioration, pain, and diverse signs of both the anterior and the posterior segments as well as abnormalities in other ophthalmic artery-supplied orbital structures [1]. The main cause of OIS is carotid artery stenosis [1]. Many OIS patients are usually undiagnosed or misdiagnosed by ophthalmologists due to the asymptomatic onset and complicated ocular manifestations. Such failures can result in irreversible blindness and an increased mortality rate [2, 3].

In the present study, we reviewed and analyzed the clinical characteristics of 42 OIS cases (53 eyes) collected from 3 different hospitals to expand the available data on OIS, to assist clinicians in differentiating OIS from other diseases and to decrease the misdiagnosis rate.

## 2. Patients and Methods

In this retrospective study, patients who presented with the clinical features of OIS or who had a history of OIS and who had visited the Department of Ophthalmology or who were referred by the Department of Neurology or Department of Cardiology were considered for inclusion. The cases were collected from three hospitals (the First Affiliated Hospital, Sun Yat-sen University; Zhongshan Ophthalmic Center, Sun Yat-sen University; and the Second Affiliated Hospital, Guangzhou Medical University) from January 2010 to December 2016. The patients of OIS were included according to the following criteria [4–6]: (1) when the stenosis of the ipsilateral (to the affected eye) internal carotid artery (ICA) was >50% and the ICA blood flow velocity was abnormal; (2) abnormal ocular symptoms and/or signs that could not be explained by other ocular diseases; and (3) fundus fluorescein angiography (FFA) with specific signs (arm-to-choroid time > 15 seconds, arm-to-retina circulation time > 18 seconds, and retinal arteriovenous time > 11 seconds). The diagnosis of OIS was established when the patient satisfied the first criterion and any two criteria in (2) or (3). The patients who were unwilling to participate or suffered from other ocular diseases, including primary glaucoma, uveitis, age-related macular degeneration, symmetrical proliferative diabetic retinopathy, choroidal detachment, retinal detachment, hereditary eye diseases, ocular tumor, or ocular trauma, were excluded. Informed consent was acquired from all of the participants before the collection of clinical materials. The study adhered to the tenets of the Declaration of Helsinki and was approved by the Institutional Review Board of the First Affiliated Hospital, Sun Yat-sen University.

All of the OIS patients underwent carotid artery color Doppler imaging (CDI) and/or computed tomographic angiography (CTA) to identify the ICA stenosis. Detailed ophthalmic examinations, including best-corrected visual acuity (BCVA), intraocular pressure (IOP), slit-lamp exam, and funduscopy were performed at each follow-up visit. The FFA (Kowa, Tokyo, Japan) was recorded in the patients who consented to the procedure and did not have any contraindications. The arm-to-choroid time, arm-to-retina time, and retinal arteriovenous time were noted, along with the presence of neovascularization and leaks. Constitutional and ocular symptoms, medical history (arterial hypertension, diabetes mellitus (DM), hyperlipidemia (HLP), coronary heart disease, cerebrovascular disease, and so on), the clinical department of the first visit, and treatments were also recorded. A statistical description was generated using SPSS for Windows, version 22.0.

## 3. Results

Forty-two OIS patients (53 eyes) were recruited in our study, including 30 males (38 eyes) and 12 females (15 eyes). Eleven patients had bilateral OIS (26.19%). The age of onset ranged from 15 to 87 years (65.10 ± 10.95), with the majority of patients aged between 61 and 75 years (29/42 = 69.50%). All of the patients had one or more systemic diseases: 37 patients had systemic arterial hypertension, 24 patients had DM, 30 patients had HLP, 24 patients had cardiovascular disease, 13 patients had cerebrovascular disease, and one 15-year-old female had Takayasu arteritis. Thirty-five patients suffered from two or more than two systemic diseases. Furthermore, 16 patients with systemic diseases were long-term smokers over 10 years. Detailed information is listed in Table 1.

Among these OIS patients, only 6 patients had ocular symptoms as their initial signs upon visiting the Department of Ophthalmology. Thirty-seven patients (88.10%) who experienced discomfort in other parts of their body first visited the Department of Neurology (28 patients) or the Department of Cardiology (9 patients). However, after making a detailed inquiry of their medical history, all 37 patients were found to have varying degrees of ocular symptoms. Only seven patients claimed they had ocular discomfort prior to constitutional symptoms, 5 patients had specific symptoms in their eyes after referral to an ophthalmologist, and 25 patients were not able to recall their exact medical history.

The ocular symptoms included amaurosis fugax (22 cases), photophobia (5 cases), visual loss (24 cases), floaters (20 cases), metamorphopsia (3 cases), phosphenes (5 cases), diplopia (2 cases), and ocular/periorbital pain (7 cases). Nevertheless, the symptoms of 20 cases of amaurosis fugax, 12 cases of floaters, and 5 cases of ocular/periorbital pain were so mild that they were discovered only after detailed inquiries. Thirty-seven patients (88.10%) complained of constitutional symptoms, such as headache, syncope, palpitations, hemiplegia, and claudication.

**Figure 1:**
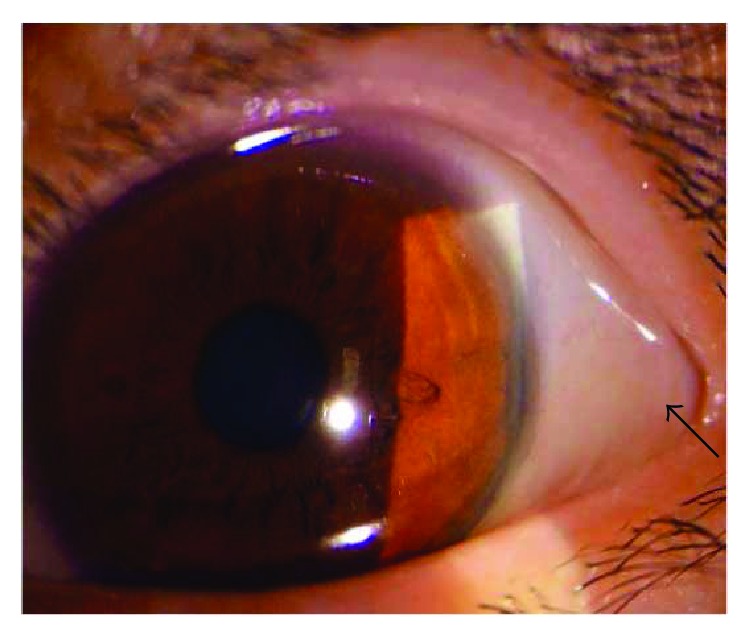
Slit-lamp photography of a 15-year-old female with Takayasu arteritis. The only pathologic finding in the anterior segment was conjunctival chemosis (arrow), but congestion was not observed. The cornea, anterior chamber, iris, and lens were normal.

**Figure 2:**
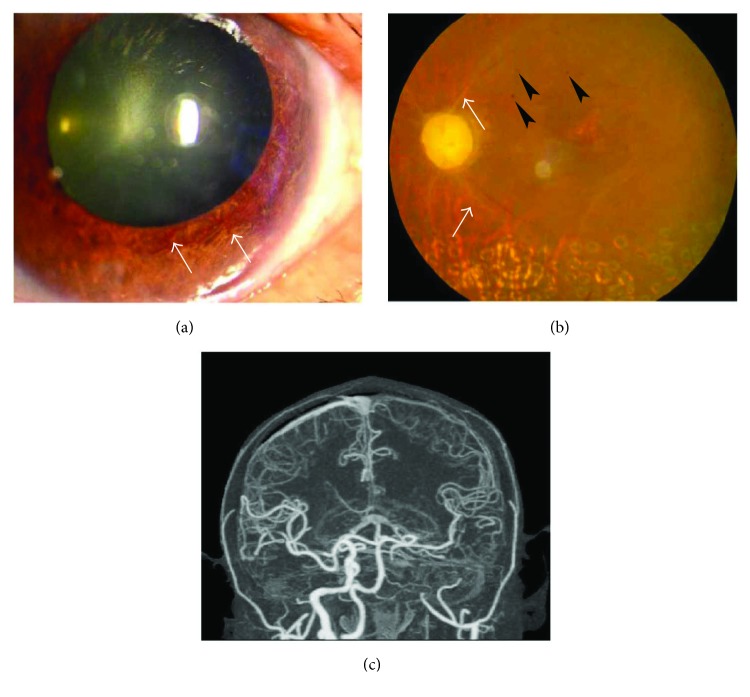
Slit-lamp photography (a), fundus photography (b), and computed tomographic angiography (CTA) (c) of a 60-year-old male OIS patient. (a) Rubeosis iridis (arrows) was found in the left eye. (b) The fundus of the left eye revealed a waxy optic disc, retinal arterial occlusion (arrows), and microaneurysms (arrowheads). This patient previously underwent retinal photocoagulation. (c) CTA demonstrated stenosis of the left ICA.

**Figure 3:**
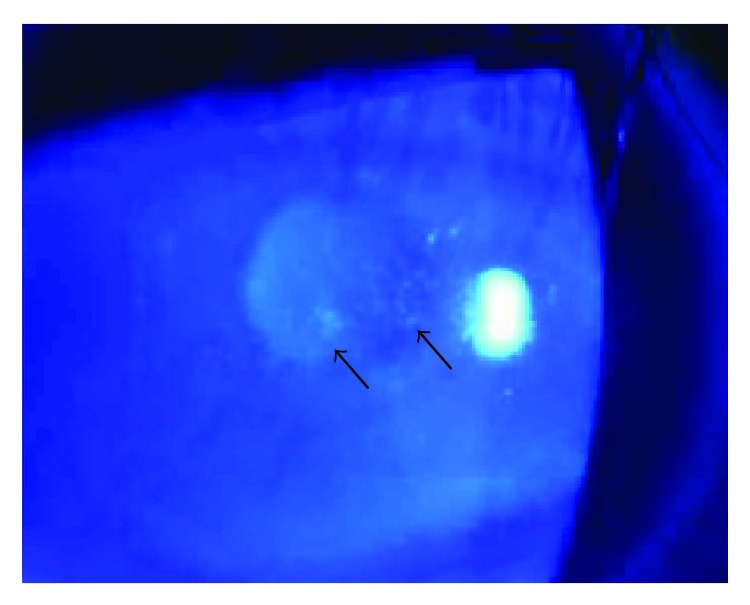
Slit-lamp photography of a 56-year-old male. Fluorescein dye revealed erosion of the corneal epithelium (arrows) under cobalt-blue light. Local superficial edema without corneal infiltration was observed around the lesion.

**Figure 4:**
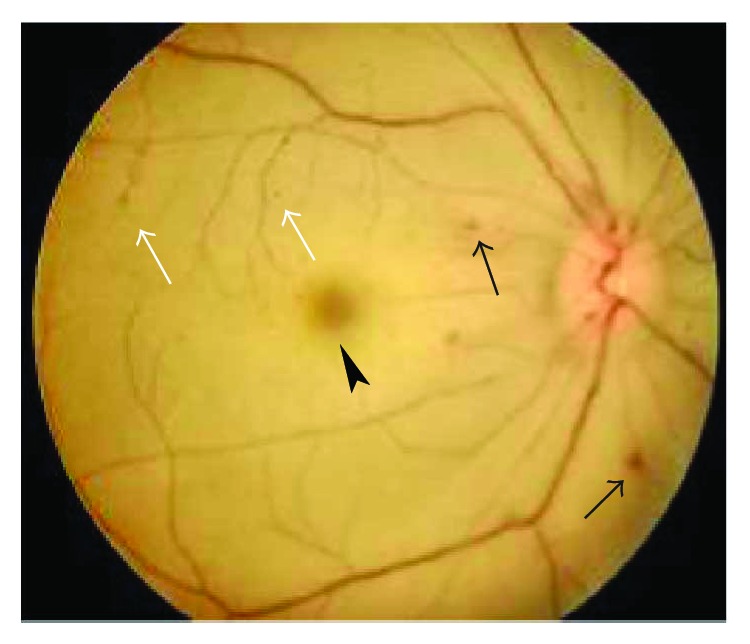
Fundus photography of a 63-year-old female. The retina of the right eye exhibited edema and was pale with punctate hemorrhages (black arrows) and microaneurysms (gray arrows). The retinal arteries became narrow, sliver-like wires, and the veins were dilated without tortuosity. A cherry-red spot (arrowhead) was found at the macula lutea.

**Figure 5:**
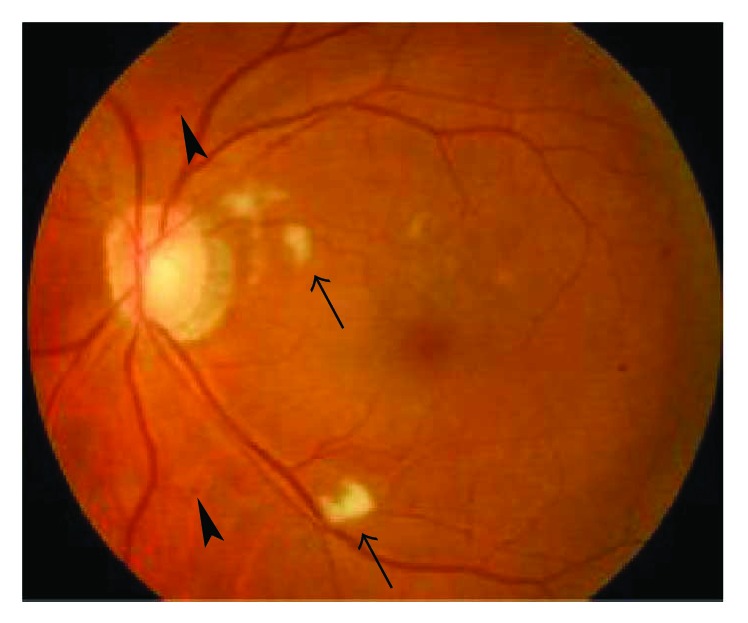
Fundus photography of a 71-year-old male. Cotton-wool spots (arrows) and microaneurysms (arrowheads) were found at the posterior pole, with narrowing arteries and dilated veins without tortuosity.

**Figure 6:**
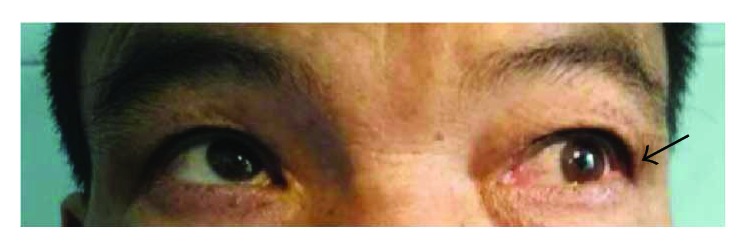
Photography of tertiary position of a 67-year-old male. The left eye had limited movement in the superotemporal direction (arrow), indicating superior rectus muscle paralysis.

The BCVA varied from light perception to 20/25, and 29 eyes (54.72%) had a BCVA of less than 20/200. The OIS patients with constitutional symptoms and a longer disease course had worse visual function. The baseline IOP was 16.74 ± 9.80 mmHg at the first visit to the ophthalmologist. Seven eyes with neovascular glaucoma had increased IOP (ranging from 25 to 47 mmHg), which was poorly controlled by glaucoma medications. Clinical signs in both the anterior and the posterior segments as well as in orbital structures were observed in OIS patients. Anterior segment signs included conjunctival chemosis (2 eyes, Figure 1), conjunctival and episcleral congestion (6 eyes), corneal edema (1 eye), corneal epithelium erosion (1 eye), iridocyclitis (3 eyes), rubeosis iridis (11 eyes, Figure 2(a)), neovascular glaucoma (7 eyes), sluggish light reflex (13 eyes), and asymmetric cataract (10 eyes). The conjunctiva was edematous without congestion or subconjunctival hemorrhage in the patients with conjunctival chemosis (Figure 1). The case of corneal epithelium erosion was revealed by fluorescence staining (Figure 3). The posterior segment abnormalities had diverse clinical manifestations. There were eight eyes with retinal edema, which exhibited narrowed retinal arterioles (5 eyes) and dilated retinal veins without tortuosity (4 eyes). Retinal hemorrhages (22 eyes) were punctate or splinter-shaped (Figure 2(b), Figure 4). Some hemorrhagic foci were easily missed on ophthalmoscopy (Figure 2(b)). Microaneurysms were found in 15 eyes (Figures 2(b), 2(c), 4, and 5). Cotton-wool spots were present in 12 eyes and always clustered at the posterior and midperipheral retina (Figure 5). Seven eyes exhibited retinal neovascularization. One eye exhibited a cherry-red spot at the macula lutea (Figure 4). Ophthalmoplegia and ptosis were the periorbital signs found in our study and were observed in one patient (Figure 6). The ocular characteristics of IOS are listed in Table 2.

**Figure 7:**
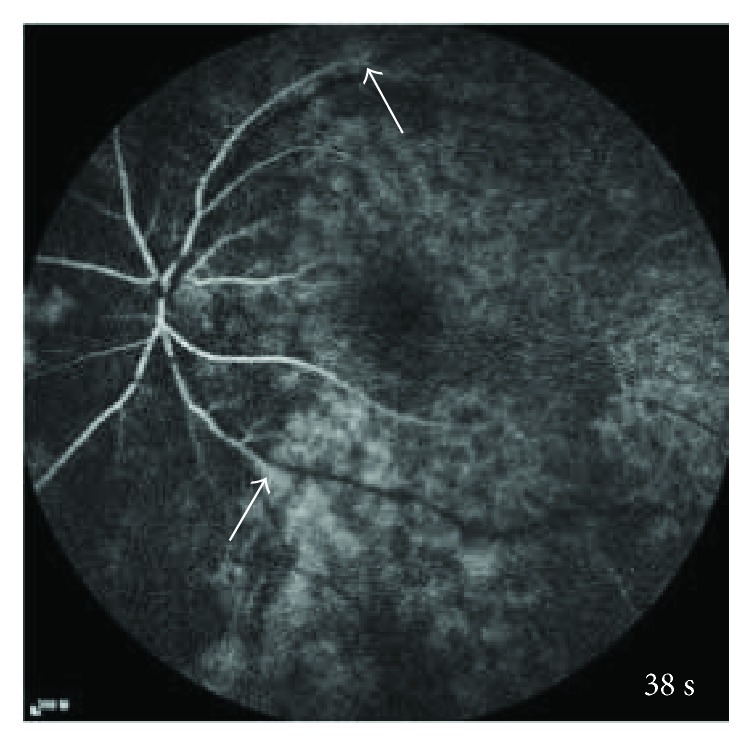
Fluorescein angiography of a 58-year-old male. The arteriovenous transit time was prolonged. At 38 seconds, the retinal arterioles are not completely filled, and the leading edge of the dye was identified (arrows). The background choroidal filling was patchy and delayed.

**Figure 8:**
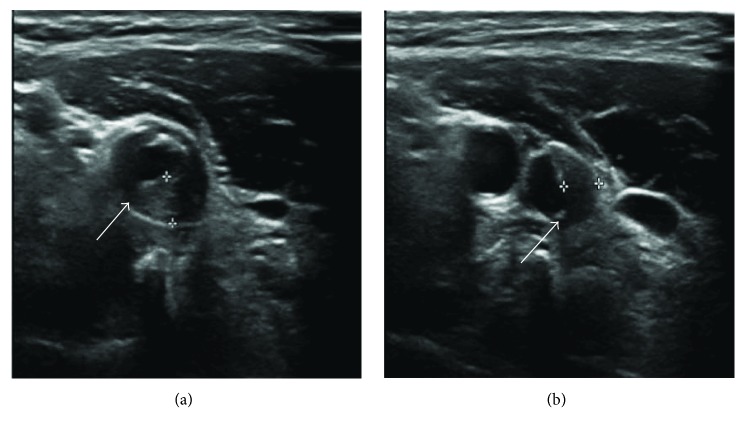
Carotid artery color Doppler imaging showed carotid artery plaques and stenosis. (a) The stenosis of the left common carotid artery is 80% (arrow). (b) The stenosis of left internal carotid artery is 60–70% (arrow).

FFA was performed in 25 patients. All of these patients had both prolonged arm-to-retina (ranging from 8.7 s to 45.5 s, median 25.0 s) and arteriovenous transit times (Figure 7). CDI and CTA revealed that among the 11 patients with bilateral OIS, 10 exhibited bilateral ICA stenosis and 1 exhibited ipsilateral ICA stenosis of the affected eye (Figures 2(c), 8(a), and 8(b)).

Among the six first-visit cases in the Department of Ophthalmology, four cases were misdiagnosed as other ocular diseases, including two that were worthy of further assessment. One case was a 56-year-old male patient who was misdiagnosed and treated for keratitis in his right eye due to superficial corneal edema and epithelium erosions (Figure 3). The patient had no signs of infection and no ocular medication or trauma history. The treatment to promote the epithelium repair was ineffective, and the keratopathy remained. The patient was euglycemic but had a medical history of systemic arterial hypertension and HLP. Thus, the diagnosis of diabetic keratopathy was excluded. At one follow-up visit, the ophthalmologist identified ipsilateral ICA stenosis of the affected eye, at which time the patient was diagnosed with OIS. The retinal examination showed very small, midperiphery hemorrhages in the right eye, which could easily be missed. Subsequent FFA revealed prolonged arm-to-choroid time and arm-to-retina circulation time. The patient's keratopathy improved markedly after a carotid endarterectomy. Another special case was a 15-year-old female with Takayasu arteritis who complained of amaurosis fugax and phosphenes (the symptoms occurred for 30 minutes when she was getting up and disappeared after she lied down), eye swelling, and tearing. At her first visit to the Department of Ophthalmology, the examination revealed conjunctival chemosis but no accompanying congestion. These findings were considered to be nonspecific features caused by anterior segment ischemia following diagnosis with Takayasu arteritis (Figure 1). For the other two cases, one patient was initially diagnosed with diabetic retinopathy due to a history of diabetes and punctate retinal hemorrhages, and one patient was diagnosed with paralytic strabismus caused by ophthalmoplegia.

## 4. Discussion

The ocular ischemic syndrome is a rare condition characterized by chronic ischemia of the anterior and/or posterior segment of the eyes and is primarily caused by the stenosis of the carotid artery. Many studies have found that the prognosis of OIS is poor [7, 8]. The mortality rate in OIS patients is up to 40% within 5 years of onset [9]. As OIS is usually asymptomatic and has poor outcomes, a strategy for establishing an early diagnosis is essential for saving visual function and improving survival.

OIS is primarily caused by stenosis of the common or internal arteries ipsilateral to the affected eye [10]. The clinical characteristics of chronic ischemia primarily occur in patients with poor collateral circulation between the two internal carotid arteries or between the internal and external carotid arteries [1]. In general, OIS is unilateral, but 20% of the cases are affected bilaterally [9, 11]. Bilateral OIS is more common found in patients with the aortic arch syndrome, hyperhomocysteinemia, and Takayasu arteritis [11–13]. The major cause of OIS is atherosclerosis [10], and other common causes include giant cell arteritis, thrombogenesis, Takayasu arteritis, trauma, and different types of diseases involving the carotid arteries [8, 14]. As the pathogenesis of atherosclerosis is associated with the male sex, advanced age, systemic metabolic diseases, and a smoking addiction, the onset of OIS is positively correlated with these factors [2, 9]. In our study, the majority of OIS patients were older males suffering from more than two systemic diseases, such as systemic arterial hypertension, DM, and HLP. There were 26.19% of patients with bilateral OIS. A 15-year-old female with bilateral OIS was suffering from Takayasu arteritis. The results of our study are consistent with the previous epidemiology studies [15, 16].

The symptoms and clinical signs of OIS are various and nonspecific. In our study, we obtained the patients' ocular symptoms after detailed inquiries. The visual dysfunction ranged from amaurosis fugax to severe visual loss. The abnormal sensations were primarily present as ocular and/or periorbital pain. Thirty-five patients (83.33%) had more than two ocular symptoms. Visual loss at varying degrees was the most common symptom. As most of the OIS patients were older, these unspecific symptoms can easily be considered the results of more common degenerative changes (e.g., presbyopia, age-related cataract, posterior vitreous detachment, and age-related macular degeneration), especially when the patient has comorbid ocular disorders or systemic diseases [17, 18].

These nonspecific symptoms and signs can share common features with other ocular diseases. The symptom of amaurosis fugax without positive abnormalities could be interpreted as a complication of anemia or postural hypotension. Patients with photophobia symptom and ocular surface changes could be underdiagnosed with dry eye, conjunctivitis, or keratitis [19]. Retinopathy and neovascularization can often be misdiagnosed with central retinal vein occlusion (CRVO) and diabetic retinopathy (DR) [1, 8]. CRVO is characterized by dilated and tortuous retinal veins, flame-shaped retinal hemorrhage, and a swollen optic disk. DR often presents with dilated and beaded retinal veins, dot and blot hemorrhages at the posterior and midperiphery of the retina, scattered microaneurysms, and hard retinal exudates. However, OIS patients present with dilated but not tortuous retinal veins and dot and blot hemorrhages at the midperipheral retina. Hard exudates are absent in OIS but present in DR. The retinal arterial perfusion pressure should be normal in CRVO and DR, but it is decreased in OIS. Another feature distinguishing OIS from CRVO and DR is that choroidal filling is delayed and patchy in OIS. Additionally, these abnormalities may appear or become more exacerbated in the context of preexisting diseases and trauma as well as previous surgery history.

In our study, one patient had unilateral corneal edema and recurrent erosions with unknown etiology. The FFA results and evidence of ICA stenosis supported the diagnosis of OIS. His lesion was cured under combined treatment with ophthalmic medicines and carotid surgery. Under the circumstance of poor blood supply from the ophthalmic artery, the corneal epithelium, corneal nerves, and tear film are affected. Ocular ischemia may also contribute to molecular alterations, such as protein and structural changes, changes in the inflammatory pathway, and altered metabolism in the cornea, effects that can mimic diabetic keratopathy. To the best of our knowledge, corneal edema and corneal epithelium erosion have not been previously considered complications of OIS.

As the rate of misdiagnosis of and failure to diagnose OIS is high, it is recommended that ophthalmologists request the detailed medical history of suspected OIS cases, especially for patients at a high risk for the condition, in order to avoid missing some of the transient symptoms, such as amaurosis, eye pain, and photophobia. Detailed ocular examinations from the anterior to posterior segment are essential in patients with unexplained conjunctival chemosis, corneal epithelium erosion, ophthalmoplegia, CRVO, or asymmetric retinopathy to exclude the possibility of OIS. OIS should also be taken into account as a differential diagnosis of uveitis, dry eye, and anterior pole ischemia syndrome, especially in unilateral cases that are nonresponsive to conventional treatment [20, 21].

As OIS is an ocular condition associated with systemic disease, patients with OIS often first visit the Department of Neurology or Department of Cardiology for constitutional symptoms. The constitutional symptoms are so prominent that OIS patients are likely to overlook the discomfort in their eyes. Consistent with this trend, only 13 of the described patients (30.95%) initially presented with ocular symptoms at the clinic. The natural course of OIS is unclear, but the prognosis and outcomes of OIS are poorer without prompt treatment. In our study, 37 OIS patients (88.10%) came to the Department of Cardiology or Department of Neurology for constitutional symptoms, but only four were referred to an ophthalmologist. We suggest that neurologists and cardiologists pay close attention to patients with confirmed or suspected carotid artery stenosis and refer these patients to ophthalmologists for further examination.

The advances of modern medicine have paradoxically hindered the diagnosis and treatment of OIS due to the increasing refinement of clinical specialties. Due to its complex, the management of OIS requires that ophthalmologists establish a cooperative relationship with cardiologists, neurologists, and interventional radiologists [5, 22, 23]. Only in this way can we maximally decrease the mortality rate and improve the quality of life of patients with OIS.

## Figures and Tables

**Table 1:** Demography and symptomatology.

Characteristic	Value (%)
Total number of patients	42
Male : female ratio	30 : 12
Onset age (yr, mean ± SD)	65.10 ± 10.95
<45	1
45~60	10
61~75	29
>75	2
Ocular symptoms^∗^	42 (100.00)
Amaurosis fugax	22 (52.38)
Photophobia	5 (11.90)
Visual loss	24 (57.14)
Floaters	20 (47.62)
Metamorphopsia	3 (7.14)
Phosphenes	5 (11.90)
Diplopia	2 (4.76)
Ocular/periorbital pain	7 (16.67)
Constitutional symptoms	37 (88.10)
Headache	20 (47.62)
Syncope	6 (14.29)
Palpitations	3 (7.14)
Hemiplegia	5 (11.90)
Claudication	3 (7.14)
Systemic diseases and living habit	
System arterial hypertension	37 (88.10)
DM	24 (57.14)
HLP	30 (71.43)
Cardiovascular disease	24 (57.14)
Cerebrovascular disease	13 (30.95)
Takayasu arteritis	1 (2.38)
Long-term smoker (>10 yrs)	16 (38.10)

^∗^Thirty-five patients (83.33%) had ≥2 symptoms.

**Table 2:** Clinical characteristics of 53 eyes with OIS.

Characteristic	Number (%)
Anterior segment	
Conjunctival chemosis (without injection)	2 (3.77)
Conjunctival and episcleral injection	6 (11.32)
Corneal edema	1 (1.89)
Corneal epithelium erosion	1 (1.89)
Iridocyclitis	3 (5.66)
Rubeosis iridis	11 (20.75)
Neovascular glaucoma	7 (13.21)
Sluggish light reflex	13 (24.53)
Asymmetric cataract	10 (18.87)
Posterior segment	
Retinal edema	8 (15.09)
Narrowed retinal arteries	5 (9.43)
Dilated retinal veins	4 (7.55)
Microaneurysms	15 (28.30)
Retinal hemorrhages	22 (41.51)
Cotton-wool spots	12 (22.64)
Cherry-red spot	1 (1.89)
Retinal neovascularization	7 (13.21)
Periorbital	
Ophthalmoplegia	1 (1.89)
Ptosis	1 (1.89)
